# Interleukin-7 levels in synovial fluid increase with age and MMP-1 levels decrease with progression of osteoarthritis

**DOI:** 10.3109/17453674.2011.645195

**Published:** 2012-02-08

**Authors:** René Rübenhagen, Jan Philipp Schüttrumpf, Klaus Michael Stürmer, Karl-Heinz Frosch

**Affiliations:** ^1^Department of Trauma Surgery, Plastic and Reconstructive Surgery, Georg-August University, Goettingen; ^2^Department of Trauma and Reconstructive Surgery, Asklepios Clinic, St. Georg, Hamburg, Germany

## Abstract

**Background and purpose:**

Little is known about biochemical mediators that correlate with the initiation and progression of knee osteoarthritis (OA). We therefore valuated the roles of cytokines and metalloenzymes in knee OA in relation to OA grading, age, and BMI.

**Patients and methods:**

A multiplex ELISA-based immunoassay (Luminex technology) was used to measure biochemical mediators in the synovial fluid (SF) of 82 patients undergoing knee surgery. All patients were classified according to age, BMI, and OA grade. 24 patients had no signs of OA (knee reconstruction surgeries). The mediators that were tested for included interleukins (IL-1Ra, IL-6, IL-7, and IL-18), chemokines (CCL2 (MCP-1), CCL3 (MIP-1a), and CXCL8 (IL-8)), growth factors (HGF and VEGF), and matrix metalloproteinases (MMP-1, MMP-2, MMP-9, and MMP-13).

**Results:**

There was a correlation between IL-7 levels in SF and age (p < 0.01). The 11 highest IL-7 levels were seen in patients who were aged between 59 and 72 but had different OA grades. In contrast, all patients who had severe OA in all 3 knee compartments (pan-OA) had only low or medium IL-7 levels. There was a negative correlation between MMP-1 levels in synovial fluid and grade of OA (p < 0.001). Correlation studies between pairs of mediators revealed two groups of mediators that are important in OA progression, dominated by MCP-1 and IL-1Ra.

**Interpretation:**

IL-7 levels in SF are elevated in elderly people suffering from OA of different grades, but they are depressed in patients with severe 3-compartment OA, possibly due to widely impaired chondrocytes embedded in the affected cartilage tissue. The observed decrease in MMP-1 levels in SF, which is dependent on the severity of OA, may be caused by deterioration of superficial cartilage layers during progression of OA.

**List of abbreviations**
BMI: body mass index
CCL: chemokine (C-C motif) ligand
CXCL: chemokine (C-X-C motif) ligand
HGF: hepatocyte growth factor
ICRS: International Cartilage Repair Society
IL: interleukin
IL-1Ra: IL-1 receptor antagonist
MCP: monocyte chemoattractant protein
MIP: macrophage inflammatory protein
MMP: matrix metalloproteinase
OA: osteoarthritis
SF: synovial fluid
VEGF: vascular endothelial growth factor

Progression of knee OA is often driven by biomechanical forces (Englund 2010), whereas the etiology of OA in other joints is less affected by mechanical stress. Biochemical mediators such as cytokines, growth factors, and matrix metalloproteinases—acting individually or in networks—profoundly influence cellular responses in joint tissues, modifying both catabolic and anabolic activities involved in the pathogenesis of OA (Goldring and Goldring 2007). Ageing is the most prominent risk factor for OA, and chondrocyte senescence and aging-related changes in the matrix, such as articular surface fibrillation and proteoglycan changes, are most likely to contribute to joint ageing (Martin and Buckwalter 2002, Shane Anderson and Loeser 2010). However, despite intensive research efforts, little is known about biochemical factors whose levels may correlate with the severity of knee OA (Belo et al. 2007). Also, age-related changes in cytokine production in body fluids have not been investigated completely (Gardner and Murasko 2002).

Biochemical mediators found in synovial fluid (SF) that affect the cellular functions of tissues of the knee joint include interleukins (ILs), chemokines, growth factors, and matrix metalloproteinases (MMPs). Interleukins, both pro- and anti-inflammatory, have a pivotal role in arthritic diseases and are potential targets of OA therapy. Chemokines, which are small, chemoattractant cytokines, have key roles in the accumulation of inflammatory cells at the site of inflammation. Growth factors produced by chondrocytes and subchondral bone regulate the growth of blood vessels in the joint. Some recent studies have supported the notion that inhibition of abnormal angiogenesis will provide effective therapeutic strategies for treatment of OA (Ashraf and Walsh 2008). MMPs and pro-inflammatory cytokines are involved in a collagen II-dependent feed-forward mechanism of matrix degradation in human articular cartilage (Klatt et al. 2009).

To improve our understanding of the molecular and cellular processes involved in joint ageing and in the initiation and progression of OA, we wanted to determine the levels of biochemical mediators that correlate with the severity of knee OA or patient age.

## Patients and methods

### Patients and their synovial fluid

Synovial fluids were collected from 82 patients who had knee surgery (total knee replacements or cruciate ligament, cartilage, or meniscal reconstruction surgeries). Patients with inflammatory or arthritic diseases other than osteoarthritis and patients who had had traumatic events 3 weeks or less before surgery were excluded. 3 patients had had traumatic events 6 weeks or less before surgery, but it is known that increased levels of cytokines caused by traumatic events revert to normal levels within 1 week (Irie et al. 2003). All patients were grouped by age and grade of OA (Kellgren and Lawrence 1957). The breakdown of age was as follows: 29 patients were aged 17–39 (mean age 29 (SD 7) years, 12 females), 28 patients were aged 40–59 (mean age 47 (SD 7) years, 8 females), and 25 patients were aged 60–82 (mean age 68 (SD 6) years, 16 females). The OA groups were as follows: 24 patients without any radiological signs of OA (OA grade 0; mean age 31 (SD 10) years, 8 females), 31 patients with OA of grade 1 or 2 (mean age 45 (SD 14) years, 14 females), and 27 patients with OA of grade 3 or 4 (mean age 63 (SD 11) years, 14 female). We obtained informed consent and written agreement from each patient. OA gradings of anterior-posterior knee radiographs were scored by an experienced observer (KHF) who was blinded to the biochemical mediator levels in the SF.

SF samples (at least 200 µL) were collected from all patients during surgery, centrifuged for 10 min at 1,000 *g* using serum monovettes (Sarstedt, Nümbrecht, Germany), and stored at –80°C until assay.

### Multiplex immunoassay

Cytokines were measured in SF samples using 3 different multiplex detection kits: *8-plex* (IL-1Ra, IL-6, IL-8 (CXCL8), IL-10, IL-17, monocyte chemoattractant protein (MCP)-1 (CCL2), macrophage inflammatory protein (MIP)-1α (CCL3), and vascular endothelial growth factor (VEGF)), *MMP-plex*(MMP-1, MMP-2, MMP-9, and MMP-13) (both from R&D Systems, Minneapolis, MN), and *4-plex* (IL-7, IL-13, IL-18, and hepatocyte growth factor (HGF); Bio-Rad Laboratories, Hercules, CA). In *MMP-plex,* total MMP was measured (pro-, active, and TIMP-bound forms). Total MMP-1 was also measured using ELISA (MMP-1 Biotrak ELISA; GE Healthcare, UK). Multiplex and ELISA assays were carried out according to the manufacturers' instructions. SF samples were diluted 4-fold in calibrator diluent or assay buffer and were measured in duplicate. Calculated coefficients of variation were below 20%, and most were below 10%. For cytokine quantification and analysis, the Bio-Plex Suspension Array System (Manager Software version 5.0; Bio-Rad Laboratories; Luminex platform) was used. Concentrations of 3 cytokines were not measurable within the dynamic range in more than 90% of samples: IL-10 (< 2.7 pg/mL), IL-13 (< 1.2 pg/mL), and IL-17 (< 2.8 pg/mL). In earlier measurements IL-1α, IL-1β, interferon-γ, and tumor necrosis factor-α (all < 2 pg/mL) were not detected in most SF samples (data not shown).

### Statistics

For correlation studies, Spearman's rank correlation coefficient was used. Statistical analysis was performed using Smith's Statistical Package version 2.80. Any p-values of < 0.05 were considered statistically significant.

## Results

### Interleukin-7

**Figure 1.**
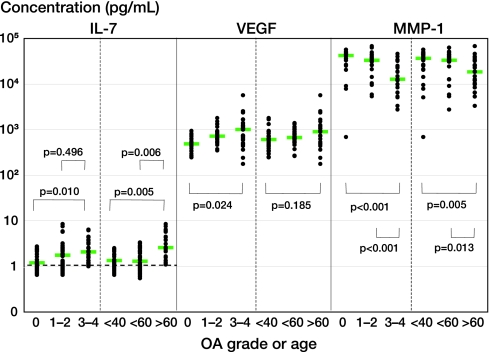
Levels of IL-7, VEGF, and MMP-1 in SF of patients classified into 3 OA-grading groups (grade 0, grade 1 or 2, and grade 3 or 4) or 3 age groups (< 40 years, 40–59 years, and > 60 years). The results from each individual are depicted. Bars indicate median concentrations. The dashed line indicates the detection limit. Significant differences between the groups are depicted by p-values; more significant p-values are written in bold.

IL-7 levels in SF correlated more with age than with OA grade (Figure 1). The median value of IL-7 doubled when comparing patients older than 60 years to those younger than 60 years (2.6 pg/mL as opposed to 1.3 pg/mL, p = 0.006).

**Figure 2.**
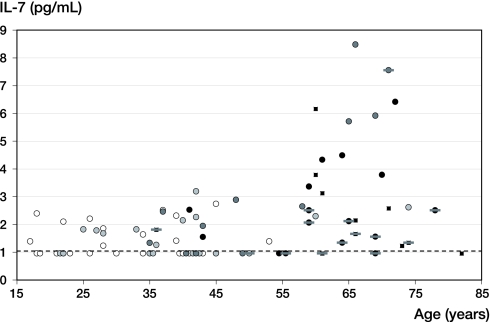
Correlation between age and IL-7 levels in SF. The results and OA grade of each individual are depicted. Grade 0: white circles. Grade 1: light-gray circles. Grade 2: dark-gray circles. Grade 3: black circles. Grade 4: black squares. The results for OA that affected all 3 knee compartments (pan-OA) are marked with a gray dash. The dashed line indicates the detection limit.

Of all patients older than 55 years, 23 had severe OA (of grade 3 or 4) (Figure 2). 11 of these 23 patients had severe cartilage lesions (ICRS grade 3 or 4) in all 3 compartments of the knee (pan-OA). Notably, all 11 of these patients had low or median IL-7 levels (< 2.55 pg/mL), but 9 of the 12 patients without pan-OA had high IL-7 levels (> 2.55 pg/mL). Spearman's rank correlation coefficient between IL-7 and patient age was 0.53 for all patients but was 0.61 for patients without pan-OA.

**Figure 3.**
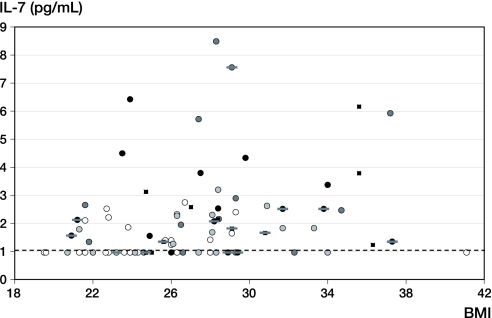
Correlation between BMI levels and IL-7 levels in SF. Spearman's rank correlation coefficient was 0.44 for all patients and 0.46 for patients without pan-OA. See the legend to Figure 2 for further details.

We also plotted IL-7 levels against BMI (Figure 3). There was a correlation between IL-7 levels and BMI, but to a lesser extent than between IL-7 levels and age. Spearman's rank correlation coefficient was 0.44, as compared to 0.53 for the correlation with age. Multiple regression analysis of IL-7 levels against age and BMI and/or grade of OA confirmed that age had a major influence on IL-7 level (p < 0.002) without having a significant influence of BMI (p ≥ 0.1) and/or grade of OA (p > 0.2).

### VEGF and MMP-1

VEGF levels in SF correlated more with OA grade than with age (Figure 1). The median value of VEGF increased 2-fold when comparing patients with OA of grade 0 to patients with OA of grade 3 or 4 (490 pg/mL vs. 1 ng/mL, p = 0.02). There was no correlation between VEGF levels and BMI (data not shown).

There was more of a negative correlation between MMP-1 levels in SF and grade of OA than between MMP-1 levels in SF and age (Figure 1). The median value of MMP-1 decreased 3-fold when comparing patients with OA of grade 0 to patients with OA of grade 3 or 4 (42 ng/mL vs. 13 ng/mL, p < 0.001). When comparing OA of grade 1 or 2 with OA of grade 3 or 4, the median MMP-1 level decreased 2.5-fold (33 ng/mL vs. 13 ng/mL, p < 0.001).

Multiple regression analysis of MMP-1 levels against grade of OA and age and/or BMI confirmed that OA grade had a major influence on MMP-1 level (p < 0.004) without having a significant influence of age (p > 0.2) and/or BMI (p > 0.2).

To validate our findings regarding MMP-1, we confirmed the results using ELISA. Here, the mean value of MMP-1 decreased 2.5-fold when comparing patients with OA of grade 0 to patients with OA of grade 3 or 4 (153 ng/mL vs. 64 ng/mL, p = 0.001). When comparing OA of grade 0 with OA of grade 1 or 2, the mean MMP-1 level decreased 1.6-fold (153 ng/mL vs. 95 ng/mL, p = 0.031). Age-related p-values were much higher (133 ng/mL vs. 68 ng/mL, p = 0.011; 133 ng/mL vs. 100 ng/mL, p = 0.21; respectively) (data not shown).

### Other mediators

**Figure 4.**
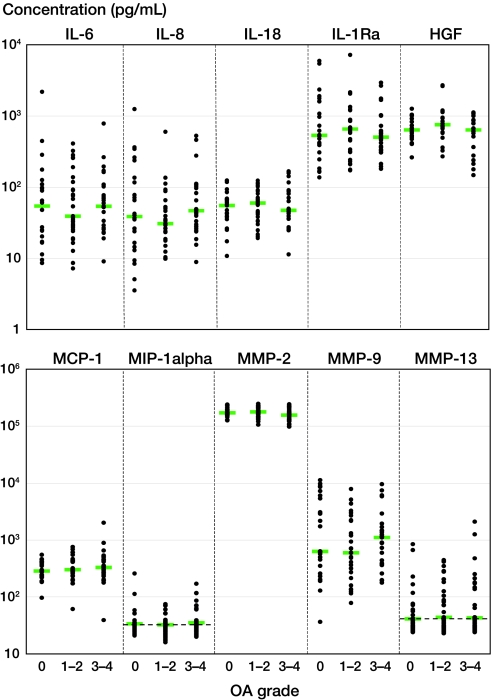
Cytokine and protease levels in SF of patients with OA grade 0, OA grade 1 or 2, or OA grade 3 or 4. The results from each individual are depicted. Bars indicate median concentrations. Dashed lines indicate detection limits. There was no significant difference in the levels of any particular mediator between groups.

The absolute levels of all other cytokines and proteases in SF showed no correlation with OA grade (Figure 4), age, or BMI (data not shown). Median values of the mediators were as follows. IL-6: 53 pg/mL; IL-8: 36 pg/mL; IL-18: 55 pg/mL; IL-1Ra: 560 pg/mL; HGF: 670 pg/mL; MCP-1: 300 pg/mL; MIP-1a: 34 pg/mL; MMP-2: 170 ng/mL; MMP-9: 720 pg/mL; and MMP-13: 43 pg/mL.

### Correlation coefficients

Table 1 (see supplementary data) summarizes the data on the relationship between the levels of 2 mediators in SF from all patients. Correlation coefficients of at least 0.5 were obtained between the level of IL-7 and age, between the levels of MCP-1 and VEGF, between the levels of IL-8 and IL-1Ra, and between the levels of IL-8 and IL-6.

In Table 2 (see supplementary data), the data from patients with OA of grade 0, 1, or 2 (Table 2a) are compared with the data from patients with OA of grade 3 or 4 (Table 2b). For patients with OA of grade 0, 1 or 2, the pairs of mediators with correlation coefficients of at least 0.5 were identical to those that were observed for all patients (compare Table 2a with Table 1). However, for grade-0, grade-1, and grade-2 patients, high correlation coefficients were obtained for BMI and IL-7 (r = 0.48) and for MMP-1 and MMP-13 or MMP-1 and IL-8 (r = 0.48 and r = 0.51, respectively). Note also that correlation coefficients between age and IL-7 decreased from 0.52 for grade-0, grade-1, and grade-2 patients to 0.06 for grade-3 and grade-4 patients (compare Table 2a and Table 2b) due to pan-OA patients with low IL-7 levels (Figure 2).

For patients with OA of grade 3 or 4, we observed 2 groups with very high correlation coefficients (Table 2b). The first group was dominated by MCP-1 (MCP-1/MMP-13: r = 0.77; MCP-1/VEGF: r = 0.64), and the second group was dominated by IL-1Ra (IL-1Ra/MMP-9: r = 0.72; IL-1Ra/IL-8: r = 0.70; IL-1Ra/MIP-1α: r = 0.65; IL-1Ra/HGF: r = 0.64). The highest increase in correlation coefficients for grade-3 and grade-4 patients compared to grade-0, grade-1, and grade-2 patients was observed for MCP-1/MMP-13 in the first group and for HGF/IL-1Ra and HGF/IL-8 in the second group (compare Table 2b and Table 2a). The correlation coefficient between MMP-1 and MMP-13 and that between MMP-1 and IL-8 decreased drastically (r = 0.48 vs. –0.26 and r = 0.51 vs. 0.14, respectively).

## Discussion

### Interleukin-7

IL-7 is a multipotent cytokine that also acts as a growth factor and is well known for its role in peripheral T-lymphocyte homeostasis (Kittipatarin and Khaled 2007). It is produced by OA chondrocytes, and IL-7-stimulated chondrocytes respond with proteoglycan release from cartilage (Long et al. 2008). The catabolic properties of IL-7 negatively affect the cartilage in at least 3 ways: inflammation-driven joint destruction, T cell-driven bone loss, and direct, harmful effects on cartilage (van Roon and Lafeber 2008).

The present study suggests that the production of IL-7 in the articular joint is strongly age-related. IL-7 levels were highly elevated in SF samples from older patients suffering from OA that did not affect all 3 compartments, suggesting that IL-7 production is part of a degenerative, age-related process that requires at least one unaffected joint compartment for elevated IL-7 production. It is known that IL-7 production is increased in cultured chondrocytes from older donors, from OA cartilage, and after stimulation with fibronectin fragments, IL-1, and IL-6 (Long et al. 2008). However, IL-7 levels were reduced in patients with 3-compartment OA (Figure 2), which could have been due to impaired cartilaginous chondrocytes unable to produce high amounts of IL-7. The loss of cartilage and chondrocytes in all 3 compartments in severe OA may finally lead to decreasing IL-7 levels in the SF. To our knowledge, this is the first observation that a cytokine level in SF was influenced differently by 1–2-compartment OA or by 3-compartment OA.

We believe that there are several possibilities for the general increase in SF IL-7 levels in elderly people (> 55 years). Ageing is accompanied by altered T-cell functions (Gardner and Murasko 2002), suggesting an effect on the expression of IL-7 from impaired chondrocytes that have lost their protective surroundings in fibrillated cartilage, which is common in elderly people (Martin and Buckwalter 2002). These age-related changes in cartilage may also directly influence IL-7 production. Furthermore, senescence of chondrocytes, which is caused by oxidative stress-induced, telomere-related genomic instability (Yudoh et al. 2005), may be the trigger for elevated IL-7 expression rates in older cartilage. Finally, other age-related changes, such as fibronectin content in the extracellular matrix, changes in proteoglycans, increased collagen crosslinking, and decreased water concentration (Martin and Buckwalter 2002) may contribute to the upregulation of IL-7 expression.

### VEGF and MMP-1

The angiogenic growth factor VEGF induces MMP expression in chondrocytes and mediates mainly destructive processes in OA. Immunostaining of OA specimens revealed that there was a correlation between the severity of OA and VEGF expression in chondrocytes, suggesting that ingrowing blood vessels may liberate pro-apoptotic signals in articular cartilage, leading to apoptotic events in OA chondrocytes (Pfander et al. 2001). Our growing understanding of the causal role of VEGF and angiogenesis in OA has been summarized by Murata et al. (2008); the expression of VEGF and subsequent VEGF-dependent signaling in OA can be viewed as a counterproductive process of the body in its attempt to repair the joint. The data from our study confirm that VEGF is an important mediator of OA progression.

MMP-1 is an interstitial collagenase that is capable of degrading interstitial collagens (types I, II, and III) and is thought to be a multifunctional molecule with important roles in diverse physiologic processes, such as development, tissue morphogenesis, and wound repair (Pardo and Selman 2005). We have shown for the first time that production of MMP-1 in the articular joint is reduced in patients with severe OA compared to patients with no or moderate OA (Figure 1). MMP-1 is mainly expressed in superficial cartilage zones, where chondrocytes deteriorate during progression of OA, while there is expression of other MMPs, e.g. MMP-9, in deeper zones (Freemont et al. 1997). In addition, we found no evidence that articular surface fibrillation, which is known to be indicative of proteolytic changes in OA cartilage, is able to increase MMP-1 expression within the cartilage, either in superficial or in deeper zones (Nguyen et al. 1992). Furthermore, we found that mechanical loading of articular cartilage reduces MMP-1 synthesis in healthy cartilage but not in OA cartilage (Monfort et al. 2006). A reduction in MMP-1 expression and activity levels is also induced by mechanical strain in fibroblast-like synoviocytes (Sun et al. 2003). Taking all these observations together, we can conclude that the reduced levels of MMP-1 in SF of patients with advanced OA may be caused by the deterioration of articular cartilage in advanced OA—due to the loss of functional chondrocytes embedded in the cartilage. In contrast to our results, no significant association with radiographic grading (Ahlbäck grading) was found when the severity of OA was compared with the levels of potential biomarkers in SF, including VEGF and MMP-1 (Anitua et al. 2009). This may have been due to the different types of OA scalings and/or methods of statistical analysis used.

### Correlations involving MCP-1 and IL-1Ra

Coefficients for correlations between levels of mediators in a body fluid may give information about the main interaction partners in the regulatory protein network of this body fluid. Changes in these correlation coefficients during the progression of OA may reflect molecular variations within signal cascades and interaction networks that are involved in the progression of the disease. In this study, we measured levels of mediators in the SF of OA patients, and correlation coefficients were compared between patients with severe OA and those with no or moderate OA (Table 2b vs. Table 2a). Our results revealed 2 groups of correlation coefficients that were high in severe OA, and MCP-1 and IL-1Ra (respectively) predominated.

In the first group, there was a strong correlation between MCP-1 levels and the levels of MMP-13 and VEGF, but there was only a correlation between MCP-1 and VEGF in the group with no or moderate OA. Besides other mediators, MCP-1, MMP-13, and VEGF were involved in progression of OA. MCP-1 has been shown to inhibit proteoglycan synthesis and to enhance proteoglycan release from chondrocytes (Yuan et al. 2001). Chondrocytes produce chemokines such as MCP-1, MIP-1a, and IL-8 that potentiate chondrocyte apoptosis and cartilage degradation in OA (Borzì et al. 2004). MMP-13 is expressed by chondrocytes and synovial cells in human OA, and is thought to play a critical role in cartilage destruction (Takaishi et al. 2008). Experiments in MMP-13 deficient mice have revealed that structural damage of cartilage in OA is dependent on MMP-13 activity (Little et al. 2009). Our results indicate that there is a dramatically increasing inter-dependence of the levels of MCP-1 and MMP-13 in SF during the progression of OA, suggesting that there is a relationship between these two mediators during the progression of the disease.

In the second group, IL-1Ra levels were highly correlated with the levels of MMP-9, MIP-1a, IL-8, and HGF, whereas IL-1Ra and IL-8 also showed a correlation in the group with no or moderate OA. The anti-inflammatory cytokine IL-1Ra has been shown to reduce cartilage degradation, MMP production, and the progression of OA lesions (Fernandes et al. 2002) and is a promising candidate for OA therapy (Calich et al. 2010). MMP-9 concentrations in the SF correlate well with the number and severity of cartilage lesions (Jouglin et al. 2000). MIP-1a acts directly on cells of the osteoclast lineage to stimulate osteoclastogenesis (Watanabe et al. 2004) and HGF may contribute to synovial neovascularization (Koch et al. 1996). Our results indicate that the correlation between the levels of IL-1Ra on the one hand and MMP-9, MIP-1a, and HGF on the other increases markedly during the progression of OA, suggesting that there is a relationship between IL-1Ra and these 3 mediators during disease progression. Expression of IL-1Ra by articular tissue cells may act as a counterbalance to OA-related phenomena such as inflammation, angiogenesis, and cartilage matrix degradation.

In summary, our data show that there is a positive correlation between IL-7 levels in SF and age, especially in patients older than 55 years. However, severe OA in all 3 compartments of the knee counteracts this age-related increase in IL-7 levels, possibly due to widely impaired chondrocytes embedded in the affected cartilage tissue. One may conclude that until this stage of cartilage deterioration, IL-7 might act as an age-dependent factor for the highly age-related progression of OA. MMP-1 levels in SF were found to decrease during progression of OA, which could be explained by diminished MMP-1 production by chondrocytes embedded in superficial cartilage layers, which deteriorate during progression of OA. VEGF was the only single mediator that showed a significant positive correlation with radiographic OA grading. However, the interconnection between several mediators was increased in severe OA, suggesting that changes in interaction patterns of biochemical mediators in SF may be more relevant for disease progression than absolute changes in the levels of individual mediators. A deeper understanding of the effects of biochemical parameters on the initiation and progression of arthritic diseases will help us to find therapeutic targets to prevent and treat OA in the future.

## Supplementary data

Tables 1 and 2 are available at our website (www.actaorthop.org), identification number 4558.

## References

[CIT0001] Anitua E, Sánchez M, de la Fuente M, Azofra J, Zalduendo M, Aguirre JJ, Andía I (2009). Relationship between investigative biomarkers and radiographic grading in patients with knee osteoarthritis. Int J Rheumatol.

[CIT0002] Ashraf S, Walsh DA (2008). Angiogenesis in osteoarthritis. Curr Opin Rheumatol.

[CIT0003] Belo JN, Berger MY, Reijman M, Koes BW, Bierma-Zeinstra SM (2007). Prognostic factors of progression of osteoarthritis of the knee: a systematic review of observational studies. Arthritis Rheum.

[CIT0004] Borzì RM, Mazzetti I, Marcu KB, Facchini A (2004). Chemokines in cartilage degradation. Clin Orthop (Suppl).

[CIT0005] Calich AL, Domiciano DS, Fuller R (2010). Osteoarthritis: can anti-cytokine therapy play a role in treatment?. Clin Rheumatol.

[CIT0006] Englund M (2010). The role of biomechanics in the initiation and progression of OA of the knee. Best Pract Res Clin Rheumatol.

[CIT0007] Fernandes JC, Martel-Pelletier J, Pelletier JP (2002). The role of cytokines in osteoarthritis pathophysiology. Biorheology.

[CIT0008] Freemont AJ, Hampson V, Tilman R, Goupille P, Taiwo Y, Hoyland JA (1997). Gene expression of matrix metalloproteinases 1, 3, and 9 by chondrocytes in osteoarthritic human knee articular cartilage is zone and grade specific. Ann Rheum Dis.

[CIT0009] Gardner EM, Murasko DM (2002). Age-related changes in Type 1 and Type 2 cytokine production in humans. Biogerontology.

[CIT0010] Goldring MB, Goldring SR (2007). Osteoarthritis. J Cell Physiol.

[CIT0011] Irie K, Uchiyama E, Iwaso H (2003). Intraarticular inflammatory cytokines in acute anterior cruciate ligament injured knee. Knee.

[CIT0012] Jouglin M, Robert C, Valette JP, Gavard F, Quintin-Colonna F, Denoix JM (2000). Metalloproteinases and tumor necrosis factor-alpha activities in synovial fluids of horses: correlation with articular cartilage alterations. Vet Res.

[CIT0013] Kellgren JH, Lawrence JS (1957). Radiological assessment of osteo-arthrosis. Ann Rheum Dis.

[CIT0014] Kittipatarin C, Khaled AR (2007). Interlinking interleukin-7. Cytokine.

[CIT0015] Klatt AR, Paul-Klausch B, Klinger G, Kühn G, Renno JH, Banerjee M, Malchau G, Wielckens K (2009). A critical role for collagen II in cartilage matrix degradation: collagen II induces pro-inflammatory cytokines and MMPs in primary human chondrocytes. J Orthop Res.

[CIT0016] Koch AE, Halloran MM, Hosaka S, Shah MR, Haskell CJ, Baker SK, Panos RJ, Haines GK, Bennett GL, Pope RM, Ferrara N (1996). Hepatocyte growth factor. A cytokine mediating endothelial migration in inflammatory arthritis. Arthritis Rheum.

[CIT0017] Little CB, Barai A, Burkhardt D, Smith SM, Fosang AJ, Werb Z, Shah M, Thompson EW (2009). Matrix metalloproteinase 13-deficient mice are resistant to osteoarthritic cartilage erosion but not chondrocyte hypertrophy or osteophyte development. Arthritis Rheum.

[CIT0018] Long D, Blake S, Song XY, Lark M, Loeser RF (2008). Human articular chondrocytes produce IL-7 and respond to IL-7 with increased production of matrix metalloproteinase-13. Arthritis Res Ther.

[CIT0019] Martin JA, Buckwalter JA (2002). Aging, articular cartilage chondrocyte senescence and osteoarthritis. Biogerontology.

[CIT0020] Monfort J, Garcia-Giralt N, López-Armada MJ, Monllau JC, Bonilla A, Benito P, Blanco FJ (2006). Decreased metalloproteinase production as a response to mechanical pressure in human cartilage: a mechanism for homeostatic regulation. Arthritis Res Ther.

[CIT0021] Murata M, Yudoh K, Masuko K (2008). The potential role of vascular endothelial growth factor (VEGF) in cartilage: how the angiogenic factor could be involved in the pathogenesis of osteoarthritis?. Osteoarthritis Cartilage.

[CIT0022] Nguyen Q, Mort JS, Roughley PJ (1992). Preferential mRNA expression of prostromelysin relative to procollagenase and in situ localization in human articular cartilage. J Clin Invest.

[CIT0023] Pardo A, Selman M (2005). MMP-1: the elder of the family. Int J Biochem Cell Biol.

[CIT0024] Pfander D, Körtje D, Zimmermann R, Weseloh G, Kirsch T, Gesslein M, Cramer T, Swoboda B (2001). Vascular endothelial growth factor in articular cartilage of healthy and osteoarthritic human knee joints. Ann Rheum Dis.

[CIT0025] Shane Anderson A, Loeser RF (2010). Why is osteoarthritis an age-related disease?. Best Pract Res Clin Rheumatol.

[CIT0026] Sun HB, Nalim R, Yokota H (2003). Expression and activities of matrix metalloproteinases under oscillatory shear in IL-1-stimulated synovial cells. Connect Tissue Res.

[CIT0027] Takaishi H, Kimura T, Dalal S, Okada Y, D'Armiento J (2008). Joint diseases and matrix metalloproteinases: a role for MMP-13. Curr Pharm Biotechnol.

[CIT0028] van Roon JA, Lafeber FP (2008). Role of interleukin-7 in degenerative and inflammatory joint diseases. Arthritis Res Ther.

[CIT0029] Watanabe T, Kukita T, Kukita A, Wada N, Toh K, Nagata K, Nomiyama H, Iijima T (2004). Direct stimulation of osteoclastogenesis by MIP-1alpha: evidence obtained from studies using RAW264 cell clone highly responsive to RANKL. J Endocrinol.

[CIT0030] Yuan GH, Masuko-Hongo K, Sakata M, Tsuruha J, Onuma H, Nakamura H, Aoki H, Kato T, Nishioka K (2001). The role of C-C chemokines and their receptors in osteoarthritis. Arthritis Rheum.

[CIT0031] Yudoh K, Nguyen T, Nakamura H, Hongo-Masuko K, Kato T, Nishioka K (2005). Potential involvement of oxidative stress in cartilage senescence and development of osteoarthritis: oxidative stress induces chondrocyte telomere instability and downregulation of chondrocyte function. Arthritis Res Ther.

