# Supplementary material for: Interleukin-7 levels in synovial fluid increase with age and MMP-1 levels decrease with progression of osteoarthritis
Source: Acta Orthop. 2012 Feb 8;83(1):59–64. doi: 10.3109/17453674.2011.645195 (PMC3278659; doi:10.3109/17453674.2011.645195)
Supplement: Supplementary file 1 [file ORT-1745-3674-83-059-s4558.pdf]

Supplementary article data

Interleukin-7 levels in synovial fluid increase with age and MMP-1 levels decrease with progression of osteoarthritis

René Rübenhagen[1], Jan Philipp Schüttrumpf[1], Klaus Michael Stürmer[1], and Karl-Heinz Frosch[2]

[1]Department of Trauma Surgery, Plastic and Reconstructive Surgery, Georg-August University, Goettingen; [2]Department of Trauma and Reconstructive Surgery, Asklepios Clinic, St. Georg, Hamburg, Germany
Correspondence: j.p.schuettrumpf@med.uni-goettingen.de
Submitted 10-12-12. Accepted 11-07-24

Table 1. Correlation (Spearman's rank correlation coefficient) between the levels of two mediators in SF from all patients

	IL-7	MMP-1	MMP-2	MMP-13	MCP-1	VEGF	IL-6	MIP-1α	MMP-9	IL-1Ra	IL-8	HGF	IL-18
Age	0.53	–0.44	–0.23	0.07	0.13	0.30	0.23	0.23	–0.02	–0.02	0.13	0.02	–0.07
BMI	0.44	–0.15	–0.06	0.01	–0.09	0.06	0.08	0.23	0.07	0.11	–0.02	0.00	0.14
IL-7		–0.04	0.17	0.31	0.12	0.24	0.26	0.44	0.02	0.04	–0.04	0.36	0.23
MMP-1	–0.04		0.38	0.18	–0.09	–0.17	0.23	0.12	–0.15	0.06	0.29	0.07	0.24
MMP-2	0.17	0.38		0.12	–0.13	–0.15	0.09	0.05	–0.24	–0.22	–0.09	0.18	0.02
MMP-13	0.31	0.18	0.12		0.40	0.39	0.24	0.12	–0.07	–0.02	0.22	0.28	0.10
MCP-1	0.12	–0.09	–0.13	0.40		0.58	0.29	0.27	0.08	0.23	0.37	0.13	–0.01
VEGF	0.24	–0.17	–0.15	0.39	0.58		0.24	0.23	0.00	0.03	0.24	0.04	0.07
IL-6	0.26	0.23	0.09	0.24	0.29	0.24		0.18	–0.16	0.36	0.50	0.22	–0.03
MIP-1α	0.44	0.12	0.05	0.12	0.27	0.23	0.18		0.32	0.33	0.32	0.23	0.24
MMP-9	0.02	–0.15	–0.24	–0.07	0.08	0.00	–0.16	0.32		0.46	0.34	–0.04	0.34
IL-1Ra	0.04	0.06	–0.22	–0.02	0.23	0.03	0.36	0.33	0.46		0.56	0.26	0.29
IL-8	–0.04	0.29	–0.09	0.22	0.37	0.24	0.50	0.32	0.34	0.56		0.06	0.34
HGF	0.36	0.07	0.18	0.28	0.13	0.04	0.22	0.23	–0.04	0.26	0.06		0.20

Open Access - This article is distributed under the terms of the Creative Commons Attribution Noncommercial License which permits any noncommercial use, distribution, and reproduction in any medium, provided the source is credited.
DOI 10.3109/17453674.2011.645195

Table 2. Correlation (Spearman's rank correlation coefficient) between the levels of two mediators in SF from patients with OA grade 0, 1, or 2 (part A) or OA grade 3 or 4 (part B)

	IL-7	MMP-1	MMP-2	MMP-13	MCP-1	VEGF	IL-6	MIP-1α	MMP-9	IL-1Ra	IL-8	HGF	IL-18
A (OA grade 0, 1, or 2)													
Age	0.52	–0.20	–0.11	0.15	0.12	0.24	0.22	0.19	–0.12	0.10	0.00	0.11	–0.06
BMI	0.48	–0.15	–0.05	0.13	–0.13	0.07	0.15	0.23	0.00	0.10	–0.09	0.06	0.25
IL-7		–0.02	0.09	0.39	0.04	0.20	0.31	0.46	0.00	0.01	–0.15	0.37	0.17
MMP-1	–0.02		0.26	0.48	0.00	0.03	0.32	0.15	–0.12	–0.02	0.51	–0.05	0.28
MMP-2	0.09	0.26		0.25	–0.10	–0.04	0.11	0.07	–0.24	–0.26	–0.06	0.22	–0.03
MMP-13	0.39	0.48	0.25		0.26	0.39	0.32	0.17	–0.03	–0.03	0.24	0.36	0.28
MCP-1	0.04	0.00	–0.10	0.26		0.54	0.21	0.23	0.06	0.17	0.37	0.09	0.11
VEGF	0.20	0.03	–0.04	0.39	0.54		0.17	0.20	–0.07	0.02	0.28	0.16	0.23
IL-6	0.31	0.32	0.11	0.32	0.21	0.17		0.12	–0.27	0.40	0.54	0.17	0.09
MIP-1α	0.46	0.15	0.07	0.17	0.23	0.20	0.12		0.30	0.31	0.29	0.19	0.30
MMP-9	0.00	–0.12	–0.24	–0.03	0.06	–0.07	–0.27	0.30		0.39	0.23	–0.13	0.33
IL-1Ra	0.01	–0.02	–0.26	–0.03	0.17	0.02	0.40	0.31	0.39		0.54	0.10	0.29
IL-8	–0.15	0.51	–0.06	0.24	0.37	0.28	0.54	0.29	0.23	0.54		–0.08	0.45
HGF	0.37	–0.05	0.22	0.36	0.09	0.16	0.17	0.19	–0.13	0.10	–0.08		0.14
B (OA grade 3 or 4)													
Age	0.06	–0.13	–0.12	–0.03	0.15	0.05	0.47	0.23	0.20	0.08	0.43	0.24	–0.05
BMI	0.11	0.26	0.13	–0.01	–0.02	–0.05	–0.10	0.18	0.22	0.15	0.05	–0.02	–0.04
IL-7		0.01	0.36	0.18	0.07	0.04	–0.04	–0.02	–0.11	0.06	0.00	0.32	0.32
MMP-1	0.01		0.46	–0.26	–0.15	–0.20	0.10	0.03	0.06	0.12	0.14	0.14	0.27
MMP-2	0.36	0.46		–0.06	–0.11	–0.04	0.12	–0.19	–0.19	–0.17	–0.01	0.03	0.08
MMP-13	0.18	–0.26	–0.06		0.77	0.54	0.22	0.43	0.05	0.28	0.38	0.27	–0.10
MCP-1	0.07	–0.15	–0.11	0.77		0.64	0.52	0.50	0.10	0.39	0.40	0.26	–0.20
VEGF	0.04	–0.20	–0.04	0.54	0.64		0.50	0.36	0.03	0.11	0.16	–0.01	–0.15
IL-6	–0.04	0.10	0.12	0.22	0.52	0.50		0.25	0.11	0.31	0.45	0.38	–0.29
MIP-1α	–0.02	0.03	–0.19	0.43	0.50	0.36	0.25		0.62	0.65	0.51	0.20	0.10
MMP-9	–0.11	0.06	–0.19	0.05	0.10	0.03	0.11	0.62		0.72	0.54	0.26	0.32
IL-1Ra	0.06	0.12	–0.17	0.28	0.39	0.11	0.31	0.65	0.72		0.70	0.64	0.28
IL-8	0.00	0.14	–0.01	0.38	0.40	0.16	0.45	0.51	0.54	0.70		0.54	0.10
HGF	0.32	0.14	0.03	0.27	0.26	–0.01	0.38	0.20	0.26	0.64	0.54		0.28
